# Aneurysm Formation at the Internal Carotid Artery Bifurcation Is Related to the Vascular Geometry of the Bifurcation

**DOI:** 10.3390/brainsci14121247

**Published:** 2024-12-12

**Authors:** Rifat Akdağ, Ugur Soylu, Özhan Merzuk Uçkun, Ömer Polat, İdris Gürpınar, Ergün Dağlıoğlu

**Affiliations:** 1 Clinic of Neurosurgery Bursa, Bursa Yüksek İhtisas Training and Research Hospital, University of Health Science, Üsküdar 34668, Türkiye; soylu.u@gmail.com; 2 Department of Neurosurgery İzmir, Medicana International Izmir Hospital, Konak 35170, Türkiye; ozhanmerzuk@gmail.com; 3 Department of Neurosurgery, School of Medicine, Duzce University, Duzce 81620, Türkiye; polatnrs@gmail.com; 4 Clinic of Neurosurgery, Ankara Bilkent City Hospital, University of Health Science, Ankara 06290, Türkiye; idrisgurpinar@gmail.com (İ.G.); edaglioglu@gmail.com (E.D.)

**Keywords:** internal carotid artery bifurcation, bifurcation aneurysm, geometry, risk factors

## Abstract

Background: In this study, we aimed to comparatively evaluate the morphology of internal carotid artery (ICA) bifurcations with and without aneurysms and identify risk factors for aneurysm development that are associated with the bifurcation geometry. Method: In this two-center study, the computerized tomography angiography data of 1512 patients were evaluated. The study included 64 (4.2%) patients with ICA bifurcation aneurysms (ICAbifAn) and patients with anterior circulation aneurysms (non-ICAbifAn). ICA (P1) was defined as the parent artery, and the middle (M1) and anterior (A1) cerebral artery segments were defined as daughter arteries. We measured the diameters of the P1, M1, and A1 and their ratios (BifSR) to identify the risk factors. In addition, we calculated the bifurcation angle in two ways by measuring all angles between the P1 and daughter arteries and compared these two methods. The first method was the angle between the M1 and A1 (α), and the second was the sum of the angles between the P1 and daughter arteries (BifA). Result: A total of 163 patients who met the inclusion criteria were included in this study: 58 patients in the ICAbifAn group and 105 patients in the non-ICAbifAn group. A univariate logistic regression analysis revealed that the P1, BifSR, α, and BifA measurements were significant predictors of aneurysm formation. However, after a multivariate analysis, only the BifA angle retained its significance (OR, 0.911 (0.877–0.946), *p* < 0.001). In the ROC curve, the optimal BifA threshold for accurately differentiating between an ICAbifAn and non-aneurysmal bifurcation was 210° (area under the curve (AUC), 0.81; sensitivity, 69%; and specificity, 87%). The α angle had an AUC of 0.68. Conclusions: These results suggest that bifurcation geometry plays a significant role in the likelihood of aneurysm formation. We also showed that the BifA was more predictive of aneurysm formation than the α angle.

## 1. Introduction

The formation of intracranial aneurysms (IAs) involves multiple factors such as environmental, anatomical, and genetic factors. Inherited traits and exposure to environmental factors such as smoking, alcohol, substance abuse, hyperlipidemia, and hypertension can increase the likelihood of aneurysm development [1,2,3,4]. Vascular morphology is another essential factor involved in aneurysm formation. The role of complex hemodynamic stresses in the vessel bifurcation area is significantly associated with the development, progression, and rupture of IAs [5,6,7,8]. Several studies have investigated the relationship between aneurysms and bifurcation morphology, and they have mostly focused on the geometry of the anterior communicating artery (ACA), middle cerebral artery (MCA), and basilar artery (BA) bifurcations [9,10,11,12,13].

The bifurcation of the internal carotid artery (ICA) occurs at the apex of the polygonal-shaped circle of Willis, and it is subjected to considerable hemodynamic stress [5,14,15,16]. Aneurysms in this region account for approximately 5% and 15% of all intracranial and ICA aneurysms, respectively. Compared to aneurysms in other regions, these aneurysms are diagnosed at smaller sizes and are prone to bleeding at younger ages due to their wider angulation and greater hemodynamic stress [16,17].

Although other bifurcation sites in the cerebral arterial tree have been extensively investigated geometrically, studies on ICA bifurcation aneurysms (ICAbifAn) have generally focused on treatment modalities and outcomes. Until now, the morphological differences between aneurysmal and non-aneurysmal ICA bifurcations have not been studied in detail. We hypothesize that the effect of ICA bifurcation geometry on aneurysm formation is similar to that of the morphological risk factors on other bifurcation regions. Thus, in this study, we aimed to comparatively evaluate the morphology of ICA bifurcations with and without aneurysms and identify risk factors for aneurysm development that were associated with the bifurcation geometry. In addition, our study aims to add valuable information to the scientific discourse by focusing on which measurements are more effective in determining the risk of aneurysm formation.

## 2. Materials and Methods

### 2.1. Study Participants and Inclusion and Exclusion Criteria

This study was based on a two-center observational database that included 1512 patients with IAs who were admitted to Bursa Medical Faculty and Ankara City Hospital between July 2016 and December 2023. Of the 1512 patients, 64 patients with 66 ICAbifAns (4.3%) were consecutively enrolled. To control genetic risk factors associated with aneurysm formation, 105 patients were included as controls (non-ICAbifAn group). The controls were randomly selected patients with aneurysms in the anterior circulation. This study was approved by the Institutional Review Board (11 August 2021, 2021/08-17). The study parameters had two main categories: demographic and radiological. Patients over the age of 18 years with a diagnosis of terminal ICAbifAn were included in the study. The following were the exclusion criteria: presence of a fusiform, dissecting, or giant aneurysm; aneurysms of the MCA-M1 segment, ACA-A1 segment, and bilateral ICA; presentation after the third day of aneurysmal bleeds; presence of aplastic A1; and poor image quality. Patients with a posterior communicating artery (PComA) aneurysm were also excluded, as a PComA aneurysm could affect the measurements of the ICA diameter and angle.

### 2.2. Demographic and Radiological Evaluation

The following demographic data were collected: age, sex, history of hypertension or diabetes mellitus, and smoking status. Computed tomography angiography and 3D reconstruction of the images were performed using a 128-slice CT (Philips Ing. Co. Philips Healthcare, Rotterdam, The Netherlands). The images were interpreted using Synapse 3D (version V4.4EU; Synapse 3D Fujifilm Medical Systems, Greenwood, SC, USA). The 3D images were constructed using maximum intensity projections and volume processing techniques with 10 mm slices. Two primary physicians measured each dataset manually, and the mean of the two measurements was used.

**Figure 1:**
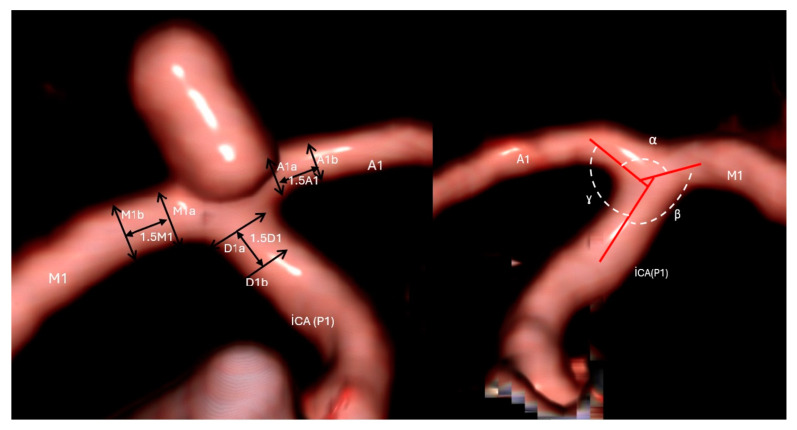
Three-dimensional CTA model of the ICA bifurcation depicting morphological variables of the surrounding vasculature. M1 = middle cerebral artery proximal segment, A1 = diameter of anterior cerebral artery proximal segment.

We analyzed the vessel diameters and angles at the ICA bifurcation, including the distal ICA (P1), MCA-M1, and ACA-A1 segments (Figure 1). The vessel diameter was determined by calculating the average of the vessel diameter (D1) directly adjacent to the aneurysm neck and bifurcation crest (D1) and the diameter of the segment located 1.5 × D cm away from the aneurysmal neck (D2) [(D1 + D2)/2]. This method was used to calculate the mean diameters of the P1, M1, and A1 segments. The bifurcation diameter-to-size ratio (BifSR) was defined as the ICA diameter divided by the sum of the M1 and A1 diameters. The M1/A1 was defined as the ratio of the M1 and A1 diameters. The ICA bifurcation angle was measured on Towne views. The angle between the M1 and A1 segments was described as α, that between the P1 and A1 segment was defined as β, and that between the P1 and M1 segment was defined as γ. The sum of the β and γ angles is defined as the bifurcation angle (BifA).

### 2.3. Statistical Analysis

We used G-Power (version 3.1.9.7; Heinrich-Heine-Universität Düsseldorf, Düsseldorf, Germany) to compute the study’s sample size. In the reference study [11], the odds ratio (OR) for SR was 0.49, requiring 140 patients for 5% alpha and 90% power. All statistical analysis was performed using SPSS for MacOS (29.0; IBM Corp., Armonk, NY, USA) and MedCalc (12.7.0.0; MedCalc Software, Ostend, Belgium). Continuous data are shown as the mean and standard deviation or the median and lowest and maximum values, whereas categorical data are shown as the frequency and percentage. The Kolmogorov–Smirnov test determined the variable normality. The independent sample *t*-test compared the groups’ normal variables. The Mann–Whitney U test compared the non-normal variables. Pearson’s chi-square test contrasted the groups’ category variables. Aneurysm risk variables were discovered using logistic regression. ROC curves were utilized to determine the thresholds and cut-off points for the angle parameters (α and BifA) with significant differences in univariate analysis. The statistical significance was set at *p* < 0.05.

## 3. Results

A total of 163 patients who met the inclusion criteria were included in this study, 58 patients in the ICAbifAn group and 105 patients in the control (non-ICAbifAn) group. The average age of the study participants was 47.6 ± 13.9 years, and 50.9% of the patients were female. There was no statistically significant difference in the age or sex between the two groups (*p* = 0.462 and *p* = 0.248, respectively). In the ICAbifAn group, 56.9% of the aneurysms were on the right side, while in the control group, 48.6% of the aneurysms were on the right side. However, this difference was not statistically significant (*p* = 0.309). No statistically significant differences were observed in the other demographic features (Table 1).

The mean ICA diameter (P1) was smaller (2.49 vs. 2.70 mm, *p* = 0.001) and the BifSR was lower (0.73 vs. 0.82, *p* < 0.001) in the ICAbifAn group than in the non-ICABifAn group. The α angle (M1–A1) was significantly larger in the ICAbifAn group than in the non-ICAbifAn group (132.8° vs. 124.4°; *p* < 0.001). However, the BifA angle was significantly smaller in the ICAbifAn group than in the non-ICAbifAn group (203.5° vs. 223.2°; *p* < 0.001). The M1/A1 did not significantly differ between the two groups (*p* = 0.620) (Table 2).

In univariate analyses, decreased BifA (OR, 0.922; 95% confidence interval [Cl] 0.898–0.947), P1 (OR, 0.213; 95% Cl, 0.084–0.539), and BifSR (OR, 0.020; 95% Cl, 0.002–0.264) were significantly associated with ICAbifAn formation. However, the α angle (OR, 1.054; 95% Cl, 1.026–1.083) was significantly inversely associated with ICAbifAn formation. Age, sex, tobacco use, hypertension, family history of hypertension, and PCoA were not statistically significant predictors of ICAbifAn formation. In the multivariate analysis, the BifA (OR, 0.911; 95% CI 0.877–0.946), BifSR (OR, 0.017; 95% CI, 0.001–0.406), and P1 (OR, 0.282; 95% Cl 0.093–0.854) were significantly associated with ICAbifAn formation (Table 3).

**Figure 2:**
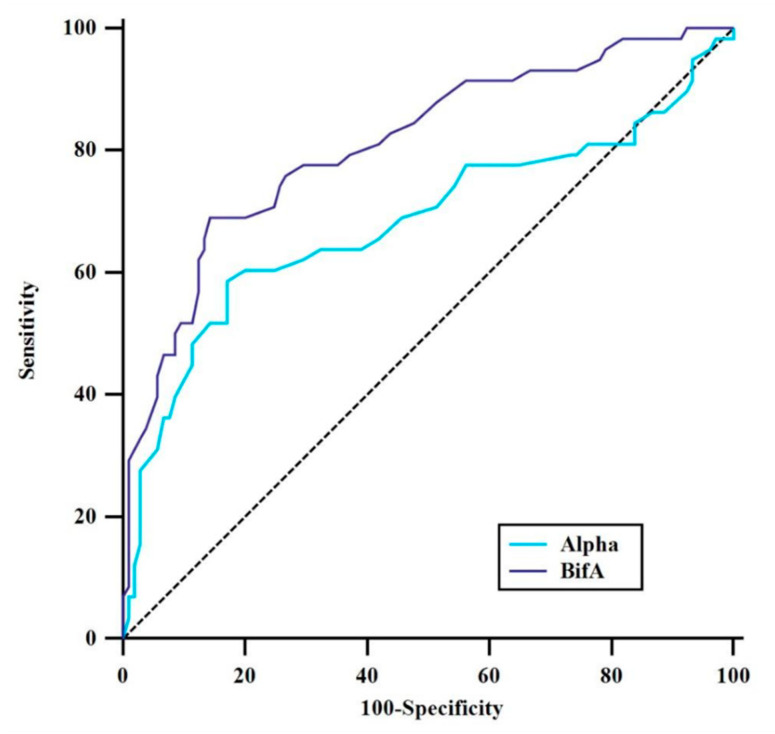
Receiver operating characteristic (ROC) curves were plotted to compare the diagnostic efficacy of the measured vascular morphological parameters α and BifA angles.

The optimal angle thresholds to distinguish between ICA bifurcations and control bifurcations were determined using ROC analysis (Figure 2). The optimal BifA threshold was 210°, with an area under the curve (AUC) of 0.81, a sensitivity of 69%, a specificity of 87%, a positive predictive value of 72.7%, and a negative predictive value of 83.3%. The cut-off α angle was 132°, which exhibited a sensitivity of 58.6%, a specificity of 82.9%, a positive predictive value of 65.4%, and a negative predictive value of 78.4% (AUC, 0.68) (Table 4). These findings indicate that the BifA was a more accurate predictor of aneurysm formation than both angles (α and BifA) that were included in the logistic regression model.

## 4. Discussion

Hemodynamic stresses play a crucial role in the formation, growth, and bleeding of IAs. The apex of a bifurcation is the area where maximum stress is experienced due to the flow impact, deflection, and flow separation [18,19,20,21,22]. Furthermore, hemodynamic stress is affected by the bifurcation geometry (diameter of the vessel and its angles). In our study, we found that the α angle (M1–A1), BifA (β + ɣ), P1, and BifSR were associated with ICAbifAn formation.

Wall shear stress (WSS) is the tangential force of friction generated by blood on the endothelium of an arterial wall. It is closely associated with the development of IAs due to arterial wall impairment. It is widely accepted that WSS is the primary hemodynamic factor that affects the development and progression of aneurysms [10,23,24,25]. The WSS generated at a bifurcation depends on the anatomy of the vessels involved, including their radius and bifurcation angle [21,25,26,27]. WSS reduces when the association between the artery diameter and bifurcation angle adheres to the principle of minimum work (PMW) [28,29]. A higher jet flow is observed at the bifurcation apex [30]. Computer simulation studies of computational fluid dynamics have shown that a smaller main artery diameter leads to a higher WSS and increased energy loss at the highest bifurcation point [30,31,32]. In our study, decreased parent artery diameter seemed to be associated with aneurysm formation and was statistically significant (OR, 0.213; 95% CI, 0.084–0.539). Our findings were consistent with studies on both MCA and BA bifurcations [11,33,34,35]. In addition, Can et al. reported in two separate bifurcation studies that a decrease in the cross-sectional area at the bifurcation site caused an increase in the blood flow velocity, which in turn initiated a change in the vascular wall, leading to a region of maximum hemodynamic stress at the top of the bifurcation [34,35]. On the contrary, according to Ćmiel-Smorzyk et al., the expansion of the main BA and MCA vessel diameter causes an increase in the volume flow rate, which in turn increases the WSS at the vessel bifurcation and initiates aneurysm formation. Furthermore, this study concluded that the increased flow rate is an independent factor associated with aneurysm formation [23]. In our study, although there was a small difference (0.21 mm) in vessel diameters between the ICAbifAn and non-ICAbifAn groups, we interpreted that the decreased ICA distal segment (P1) diameter caused increased WSS by affecting the PMW in the bifurcation region, thus affecting aneurysm formation.

The BifSR, which is the ratio of the parent artery’s diameter to the combined diameter of the bifurcation branches, is not influenced by the size of the aneurysm [6,11]. Therefore, the relationship between the diameters of the ICA distal segment P1, MCA proximal segment M1, and ACA proximal segment A1 vessels forming the ICA bifurcation may be a robust measure of aneurysm formation. The flow within the bifurcations in the cerebral arterial vascular tree depends on various morphological features, including the relative caliber and angulation of the parent and daughter branches. This is also believed to follow the PMW. Based on this theory, the diameter of the main artery provides a constant WSS, while the diameters of its smaller daughter branches are among the determinants of the final WSS at the bifurcation region [36,37]. Both a comprehensive study on MCA bifurcation aneurysms, one of the most common sites of intracranial aneurysms, and a recent study on BA bifurcation by Zhang et al. have shown that decreased BifSR increases the risk of aneurysm formation [6,11]. Conversely, a recent study reported that the dimensions of the vessels forming the circle of Willis do not conform to the PMW; they may follow a yet undetermined optimality principle. In this comparative study conducted with MCA and BA bifurcations, they stated that aneurysm formation depends on the presence of asymmetric daughter vessel diameters rather than the ratio of vessel diameters to each other [23]. However, the dimensions of the intracranial arteries beyond the circle of Willis conform to the optimality principle (especially the MCA bifurcation) [23,38]. In our study, the BifSR was significantly lower in the ICAbifAn group than in the non-ICAbifAn group, indicating a violation of the vascular PMW. This means that the vascular diameter ratios of the aneurysmal ICA bifurcation do not follow the PMW, which may lead to higher hemodynamic stress and aneurysm formation at the ICA bifurcations.

The presence of aneurysms in IA bifurcations is associated with large bifurcation angles in both the MCA and BA bifurcations, which are anatomically the terminal bifurcation of a large feeder artery and therefore similar to ICAbif [9,10,11,18,33,36]. In particular, BA is similar to ICA bifurcation because it is a T-type bifurcation. The relationship between the vessel wall biology and the hemodynamics forces is a key part of how aneurysms develop. This is because they are hypothesized to be linked to endothelial cells that respond destructively to high WSS [39]. Lauric et al. have shown that narrow bifurcations (α angle in our study) are mostly characterized by a protective WSS gradient, while bifurcations with wide and asymmetric vessels are exposed to an aneurysogenic positive WSS gradient. Thus, this destructive effect on the vascular walls causes remodeling [36]. Recent animal studies with experimental models have also demonstrated a positive association between the bifurcation angulation and aneurysm formation and growth [40,41]. In a multicenter case–control study conducted by Shimuzi et al., it was shown that a bifurcation angle of ≥145° was associated with a 5.5-fold increase in aneurysm progression compared to a bifurcation angle of less than 145° [41]. However, in a multicenter prospective study, Boucherti et al. stated that bifurcation angles may increase due to IA formation [42]. In a study of 183 cases, Li et al. reported that the geometry between the BA and the posterior cerebral artery was not associated with aneurysm progression or rupture [43]. In another recent anatomy study of BA aneurysms, they showed that the angulation of the parent artery and its branches had effects on aneurysm formation [44]. In parallel with many radiological and experimental studies, our findings showed the existence of a relationship between the vascular angulations that form the ICA bifurcation geometry and aneurysm formation.

The determination of bifurcation angles in the literature greatly varies. While some studies are based on the angulation between both daughter vessels [11,18], other studies have used the sum of the angulations between the parent and daughter vessels [9,10,45,46]. In our current study, we evaluated both measurements and determined that both measurements were associated with the formation of ICAbifAn. Statistically, when we examined the effect of α angle and BifA, we showed that the α angle approached significance [OR, 1.054 (1.026–1.083), *p* < 0.001]; however, only the BifA angle retained significance after the multivariate analysis [OR, 0.911 (0.877–0.946), *p* < 0.001]. When these two angles, which are determinants of aneurysm formation, were subjected to ROC analysis to determine the cut-off values with optimum sensitivity and specificity, the largest AUC value of 0.81 was observed for the BifA angle, while the AUC value of the α angle was found to be 0.68. This shows that the BifA angle is a more accurate predictor of aneurysm formation than the alpha angle. The optimal cut-off values to most accurately distinguish aneurysmal from non-aneurysmal bifurcations were found to be 210° for BifA (sensitivity 0.69, specificity 0.87) and 58.8° for the alpha angle (sensitivity 0.58, specificity 0.82) (Figure 2). The reason for this difference may be the smaller angles between the parent artery and the daughter arteries (daughter arteries originating from the parent artery with sharper angles). Because the flow at the apex of the bifurcation is steep and divides into branches after the crossover, it tends to be laminar as it progresses toward the branches and reaches the highest hemodynamic stress in the small-angle artery, preceded by chaotic turbulence. This chaotic environment causes wall thickening and intimal hyperplasia in the arterial wall and, as a result, may cause aneurysm formation [21,47]. Therefore, when evaluating the bifurcation geometry, we think that evaluating the sum of the angles between the parent artery and the daughter branches (BifA) will be more useful than evaluating the angles between the branches (α).

Our study has some limitations. The retrospective nature of our study was the primary limitation. Furthermore, we cannot say with certainty that bifurcation geometry has an effect on aneurysm formation. This is because the development of intracranial aneurysms probably also depends on a large number of variables (such as biological, biochemical, and genomic data) that determine changes in vasculopathy that were not fully considered in this study. Another limitation of our study was the lack of available morphologic data before aneurysm formation. Another limitation of our study is that subgroup analysis could not be performed due to the low number of small aneurysms (≤3 mm). Although the effect on the results is small, the measurements were performed manually. This is a much more feasible technique in clinical practice, but the bias in measurements between investigators cannot be ignored. The active use of artificial intelligence, more detailed examinations of arterial walls with high Tesla MRI, and combinations of methods such as machine learning techniques that can perform automatic measurements can reduce such biases in risk assessment.

## 5. Conclusions

In this study, we evaluated the effects of the adjacent bifurcation geometry on the formation of ICAbifAn. To minimize the genetic risk factors, we included patients diagnosed with anterior circulation aneurysms as a control group. Our study findings demonstrate that the risk of aneurysm formation increases as the α angle increases and the BifA, P1, and BifSR decrease. Furthermore, BifA appears to be a better predictor of aneurysm formation than the α angle. These findings support our hypothesis that the bifurcation geometry may significantly impact the risk of aneurysm formation. Therefore, clinicians can quickly and easily perform basic morphologic measurements on 3D reconstructed images of the ICA bifurcation in high-risk patients, such as those with previous subarachnoid hemorrhage or family history, to aid risk assessment.

## Figures and Tables

**Table 1:** Demographic data and clinical risk factors of patients with and without ICAbifAn. SD = standard deviation, ICAbifAn = internal carotid artery bifurcation aneurysm.

Characteristics	Total (n = 163)	ICAbifAn (n = 58)	Non-ICAbifAn (n = 105)	*p*-Value
Age, (years) (SD)	47.6 ± 13.9	48.5 ± 14.3	47.1 ± 13.7	0.462
Sex, n (%)				0.248
Female	83 (50.9)	26 (44.8)	57 (54.3)	
Male	80 (49.1)	32 (55.2)	48 (45.7)	
Hypertension, n (%)	81 (49.7)	33 (56.9)	48 (45.7)	0.172
Smoking, n (%)	56 (34.4)	22 (37.9)	34 (32.4)	0.475
Family, n (%)	18 (11)	7 (12.1)	11 (10.5)	0.960
Side, n (%)				0.309
Right	84 (51.5)	33 (56.9)	51 (48.6)	
Left	79 (48.5)	25 (43.1)	54 (51.4)	

**Table 2:** Characteristics of the surrounding vasculature for ICAbifAn and non-ICAbifAn. SD = standard deviation, P1 = diameter ICA distal segment, M1 = diameter of middle cerebral artery segment 1, A1 = diameter of anterior cerebral artery segment, PComA = posterior communicating artery, ICAbifAn = internal carotid artery bifurcation aneurysm.

Characteristics		Total (N = 163)	ICAbifAn (n = 58)	Non-ICAbifAn (n = 105)	*p*-Value
M1–A1 angle in degrees (α) (Min–Max)		127 (96–155)	135 (96–155)	125 (100–155)	<0.001
Sum of ICA-M1 (β) and ICA-A1 (ɣ) angles (BifA) (SD)		216.2 ± 17.6	203.5 ± 16.5	223.2 ± 14	<0.001
ICA diameter in mm (P1) (SD)		2.62 ± 0.37	2.49 ± 0.42	2.70 ± 0.32	0.001
M1/A1 diameter ratio		1.56 ± 0.56	1.59 ± 0.50	1.55 ± 0.59	0.620
Diameter size ratio (SR) P1/(M1 + A1) (SD)		0.79 ± 0.16	0.73 ± 0.14	0.82 ± 0.17	<0.001
PComA, n (%)	Yes	111 (68.1)	36 (62.1)	75 (71.4)	0.293
	Fetal	16 (9.8)	6 (10.3)	10 (9.5)	0.858

**Table 3:** Univariable and multivariable logistic regression for the presence of an ICAbifAn.

Characteristics	Univariate		Multivariate	
	Odds Ratio (95% CI)	*p*-Value	Odds Ratio (95% CI)	*p*-Value
Age	1.007 (0.984–1.031)	0.549		
Sex: Male	1.462 (0.767–2.784)	0.248		
Hypertension	1.567 (0.821–2.991)	0.173		
Smoking	1.276 (0.653–2.493)	0.475		
Family	1.173 (0.428–3.211)	0.756		
Side: Right	0.715 (0.375–1.364)	0.309		
M1–A1 angle in degrees (α)	1.054 (1.026–1.083)	<0.001	0.977 (0.937–1.018)	0.265
Sum of P1–M1 (β) and P1–A1 (ɣ) angles (BifA)	0.922 (0.898–0.947)	<0.001	0.911 (0.877–0.946)	<0.001
ICA diameter in mm (P1)	0.213 (0.084–0.539)	0.001	0.282 (0.093–0.854)	0.025
M1/A1 diameter ratio	1.136 (0.638–2.023)	0.666		
Diameter size ratio (BifSR) P1/(M1 + A1)	0.020 (0.002–0.264)	0.003	0.017 (0.001–0.406)	0.017
Pcom: Fetal	0.818 (0.259–2.589)	0.733		

P1 = diameter İCA distal segment, M1 = diameter of middle cerebral artery segment, A1 = diameter of anterior cerebral artery segment, PCoA = posterior communicating artery, ICAbifAn = internal carotid artery bifurcation aneurysm, Cl = confidence interval.

**Table 4:** Receiver operator characteristics (ROC) plot showing the performance of α and bifurcation angles (BifA) in discriminating between aneurysmal and non-aneurysmal internal carotid cerebral artery (ICA) bifurcations. P1 = diameter İCA distal segment, M1 = diameter of middle cerebral artery segment, A1 = diameter of anterior cerebral artery segment. Cl = confidence interval.

Risk Factor	AUC (95% CI)	Cut-Off	*p*-Value	Sensitivity (%)	Specificity (%)	PPV (%)	NPV (%)
M1–A1 angle in degrees (α)	0.682 (0.586–0.778)	>132	<0.001	58.6	82.9	65.4	78.4
Sum of ICA-M1 (β) and P1–A1 (ɣ) angles (BifA)	0.810 (0.739–0.882)	≤210	<0.001	69.0	85.7	72.7	83.3

## Data Availability

Data supporting the findings of this study are available upon reasonable request from the corresponding author, Rifat Akdağ. The data are not publicly available due to confidentiality and ethical constraints.
